# Nucleotide Context Can Modulate Promoter Strength in Genes Transcribed by RNA Polymerase III

**DOI:** 10.3390/genes14040802

**Published:** 2023-03-27

**Authors:** Danil V. Stasenko, Karina A. Tatosyan, Olga R. Borodulina, Dmitri A. Kramerov

**Affiliations:** Laboratory of Eukaryotic Genome Evolution, Engelhardt Institute of Molecular Biology, Russian Academy of Sciences, 119991 Moscow, Russia

**Keywords:** RNA polymerase III, promotor, transcription, non-coding RNA, SINE, transfection, *Mus musculus*

## Abstract

The small nuclear RNAs 4.5SH and 4.5SI were characterized only in mouse-like rodents; their genes originate from 7SL RNA and tRNA, respectively. Similar to many genes transcribed by RNA polymerase III (pol III), the genes of 4.5SH and 4.5SI RNAs include boxes A and B, forming an intergenic pol III-directed promoter. In addition, their 5′-flanking sequences have TATA-like boxes at position −31/−24, also required for efficient transcription. The patterns of the three boxes notably differ in the 4.5SH and 4.5SI RNA genes. The A, B, and TATA-like boxes were replaced in the 4.5SH RNA gene with the corresponding boxes in the 4.5SI RNA gene to evaluate their effect on the transcription of transfected constructs in HeLa cells. Simultaneous replacement of all three boxes decreased the transcription level by 40%, which indicates decreased promoter activity in a foreign gene. We developed a new approach to compare the promoter strength based on the competition of two co-transfected gene constructs when the proportion between the constructs modulates their relative activity. This method demonstrated that the promoter activity of 4.5SI is 12 times that of 4.5SH. Unexpectedly, the replacement of all three boxes of the weak 4.5SH promoter with those of the strong 4.5SI gene significantly reduced, rather than enhanced, the promoter activity. Thus, the strength of a pol III-directed promoter can depend on the nucleotide environment of the gene.

## 1. Introduction

In contrast to RNA polymerase II (pol II) largely synthesizing mRNA and various long non-coding RNAs, RNA polymerase III (pol III) synthesizes small RNAs. These include 5S rRNA, all tRNA species, 7SL RNA (a component of the signal recognition particle), 7SK RNA (a factor of pol II transcription elongation), U6 RNA (a component of spliceosomes), Y RNA (a factor of initiation of DNA replication), and vault RNA (a regulator of cellular autophagy), etc. [1,2,3,4,5]. Pol III also transcribes mobile genetic elements referred to as SINEs (short interspersed elements). These elements, largely from 150 to 300 bp in length, originate from tRNA species and more rarely from 5S rRNA or 7SL RNA [6,7].

**Figure 1:**
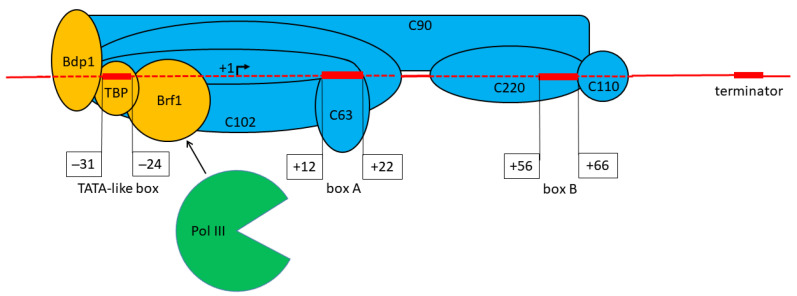
A complex of TFIIIB and TFIIIC with a gene carrying a type 2 promoter for pol III. The subunits of TFIIIB and TFIIIC are colored yellow and blue, respectively. Pol III (green) is shown as disproportionately small and without subunits. The TSS is indicated as +1. The TATA-like box, box A, box B, and transcription terminator are shown as red rectangles; the positions of the boxes relative to the TSS are specified.

Three types of pol III-directed promoters are known [2,3,8]. The last described type 3 promoter resides in the 5′-flanking region of the gene, thus resembling the pol II promoters. Type 3 promoters are typical for the selenocysteine tRNA, 7SK RNA, U6 RNA, Y RNA, and certain other genes. The type 1 and 2 promoters completely or largely reside within the transcribed DNA sequence and are referred to as internal or intergenic promoters. Type 1 promoters are found only in the 5S rRNA genes and derived SINEs; they are composed of three regions (boxes A and C, as well as the intermediate element) at some distance apart from each other. Type 2 promoters mediate the transcription of tRNA, 7SL RNA, vault RNA, BC1 and BC200 RNAs, adenovirus VA-I and VA-II RNAs, and EBER1 and EBER2 RNAs of Epstein-Barr virus. Apart from that, such promoters are found in tRNA- and 7SL RNA-derived SINEs. The latter type is the subject of this study. The type 2 promoter consists of two 11 nt sequences called boxes A and B. Box A is 11 bp from the transcription start site (TSS), and box B is 30–40 nt downstream of the box A (Figure 1). The boxes notably differ between genes; however, their consensus sequences can be deduced, e.g., from the human tRNA genes and various SINEs: TRGYnnARBGG (box A) and GGTTCRAnYCY (box B), where Y = C or T; R = G or A; and B = T, C, or G [7,9]. The transcription factor IIIC (TFIIIC), composed of five subunits, recognizes and binds boxes A and B (Figure 1). Due to protein–protein interactions, this complex binds the transcription factor TFIIIB, which is composed of three subunits: TBP, Brf1, and Bdp1; as a result, TFIIIB comes close to the upstream sequence of the gene. Finally, pol III is recruited due to the interaction of the 17-subunit RNA polymerase with Brf1 [8,10].

In addition to boxes A and B, the tRNA genes of plants and some yeasts (*S. pombe*) have a TATA box or a very similar sequence in their 5′-flanking regions at position −30/−24 [11]. The TATA box promotes TFIIIB recruitment via direct TBP binding to the DNA signals. The tRNA genes of most animals usually have no TATA boxes, and the sequences at the corresponding positions (around −30/−24) have little resemblance to a TATA box [11,12,13]. Other non-coding RNA genes with a type 2 promoter lack a canonical TATA box, but the corresponding region (−30/−24) can contain TATA-like sequences [14,15,16,17,18,19,20]. Unlike canonical TATA boxes, such TATA-like boxes cannot bind TBP [21,22]. Modifications of such TATA-like boxes towards GC-rich sequences proved to dramatically reduce gene expression, which indicates the significance of these boxes for pol III transcription [14,18,20,23].

The subjects of this research were murine genes of two small nuclear RNAs (4.5SH and 4.5SI) possessing type 2 promoters [24,25]. The 4.5SH and 4.5SI RNAs were found and sequenced a long time ago [26,27]. Although these RNAs have similar lengths (94 and 98 nt, respectively), their nucleotide sequences are not similar. The 4.5SI RNA is present in mouse-like rodents: mice, rats, gerbils, hamsters, voles, and bamboo and mole rats [25]. The 4.5SH RNA is characteristic of the same rodents, as well as jerboas and birch mice, which indicates its earlier appearance in evolution [24].

The genomes of mice and rats have three 4.5SI RNA genes located 4–40 kb from one another [28]. There are hundreds of 4.5SH RNA gene copies in the genomes, and each gene is part of a long (4–5 kb), tandem repeat [24,29]. The nucleotide sequences of the 5′-terminal region of 4.5SI RNA and tRNA-derived B2 SINE are substantially similar; moreover, the distribution of B2 and 4.5SI RNA coincides in rodents [24,30,31]. Apparently, the emergence of this SINE family and 4.5SI RNA is tightly linked in the evolution of rodents. The nucleotide sequence of 4.5SH RNA shows a clear similarity with that of B1, another rodent SINE [30,32]. This family of SINEs, typical for all rodents [33], originates from 7SL RNA [34]. In all likelihood, the 4.5SH RNA gene emerged in the common ancestor of mouse-like rodents from the ancient B1 subfamily pB1d10 [32].

A large number of 4.5SH and 4.5SI RNA molecules can be found in various cells and tissues of mouse-like rodents, localized predominantly in the cell nuclei [35,36]. 4.5SI RNA is stable in the cell, whereas 4.5SH RNA is characterized by rapid turnover [37]. The cellular level of 4.5SH RNA increases significantly upon heat shock exposure [38]. The function of 4.5SI RNA is still unknown. Until recently, the role of 4.5SH RNA also remained unclear; however, new experiments have demonstrated that 4.5SH RNA functions as a molecular antidote for toxic exonization of antisense SINE B1 insertions in introns of genes [39].

Previously, we have identified a TATA-like box at position −31/−24 required for the efficient transcription of these genes by transfecting HeLa cells with the 4.5SH and 4.5SI RNA genes with deletions in the 5′-flanking sequences [20,23]. The nucleotide sequences of the TATA-like boxes are dissimilar in these two genes. Nucleotide substitutions were introduced to identify nucleotides significant for the efficient transcription at each position of the box [20]. The pattern of the most significant nucleotides differed notably in these two genes, which suggested a specific function of these TATA-like boxes. In addition, specific features of the A and B boxes in 4.5SH and 4.5SI RNA genes were not surprising considering their different origins. These findings prompted us to test the efficiency of A, B, and TATA-like boxes transferred from one gene to another. In addition, we replaced certain unconventional nucleotides in boxes A and B with conventional ones. Our experiments demonstrated that such changes in the pol III-directed promoter had either no effect or reduced transcription efficiency. The reduced promoter strength activity was specifically exposed by the proposed transcriptional competition method. This method was used to demonstrate that the promoter of the 4.5SI RNA gene is 12 times stronger than that of the 4.5SH RNA gene. However, the simultaneous replacement of all three boxes of the weak 4.5SH promoter with the corresponding 4.5SI boxes notably reduced, rather than increased, promoter strength. These data indicate that the nucleotide environment of the gene can contribute to promoter strength. This result contradicts current views on boxes A and B, as well as the TATA-like box (if any), as the only sequence factors of pol III-dependent transcription (see reviews [2,3,8,10]). The data obtained indicate that the nucleotide context can indirectly modulate the function of type 2 promoters. Alternatively, certain nucleotides missing in the three boxes can interact with the transcription factors and thus impact the promoter strength. Notice the convenience of the 4.5SI and 4.5SH RNA genes for such studies. They have a relatively narrow distribution (mouse-like rodents), which facilitates monitoring the transcripts of these genes introduced into other mammalian cells without interfering with similar endogenous RNAs. 

## 2. Materials and Methods

### 2.1. Plasmid Constructs

The cloning of the murine 4.5SI (the Mmu’ copy) and 4.5 SH RNA genes into the pGEM-T vector was described elsewhere [20,23,28]. The constructs with the wild-type genes and the 32-bp 5′-flanking genomic sequences (SIwt and SHwt) were generated and used for mutation analysis previously [20]. All constructs with nucleotide substitutions were obtained using the Phusion Site-Directed Mutagenesis Kit (Thermo Fisher Scientific, Waltham, MA, USA) and the SIwt or SHwt plasmids as the PCR template. This method preserves the initial insertion orientation, which is critical to adequately compare the transcription efficiency of different constructs, considering the possible effect of the flanking vector sequences [23,40]. The constructs B1_6 and B2_2 containing corresponding SINE copies with truncated 3′-terminal sequences were described previously [20]. A DNA fragment synthesized using a random sequence generator (Section 3.1) was ligated into pGeneClip (Promega, Madison, WI, USA) to yield pU1. The ballast (empty) pGEM-T was generated via the self-ligation of a considerable amount of the plasmid (which yields circular pGEM-T in the absence of a PCR-generated DNA). Clones of all new constructs with substitutions of interest and without spontaneous mutations were grown, and plasmids were isolated using the Plasmid Midi Kit (Qiagen, Hilden, Germany) according to the manufacturer’s protocol.

### 2.2. Cell Transfection and Northern Blot Analysis

HeLa cells (ATCC, CCL-2) were grown to an 80% confluent monolayer in 60 mm Petri dishes using DMEM with 10% fetal bovine serum. Cells on one dish were co-transfected with 4 μg of plasmid DNA (SHwt, SIwt, or their derivatives) and 1 μg of the control plasmid pU1 mixed with 10 μL of TurboFect transfection reagent (Thermo Fisher Scientific, Waltham, MA, USA) according to the manufacturer’s protocol. Transcriptional competition experiments were conducted in a similar way but without pU1. Cells on each plate were co-transfected with different quantities (specified in figures in Section 3.3) of a target (0.25 μg of SIwt or 4 μg of SHwt) and competitor constructs. Their mixture was supplemented with the ballast pGEM-T to a total of 5 µg. All transfections were conducted in three replicates. In this work, we used human cells (HeLa) for transfection because, unlike any mouse cells, they lack endogenous 4.5SH and 4.5SI RNA. Accordingly, HeLa cells are suitable for monitoring the transcription of these mouse genes. The cellular RNA was isolated 20 h after transfection using the guanidine-thiocyanate method [41].

RNA samples (10 μg) obtained from each transfection were separated via electrophoresis in a 6% polyacrylamide gel with 7 M urea, transferred to a nylon membrane (GVS, Bologna, Italy) using semi-dry electroblotting, and hybridized with probes labeled using (^32^P)dATP, Taq-polymerase, and reverse primers. The labeling conditions, primers, and 4.5SH and 4.5SI RNA probes, as well as the Northern hybridization conditions, were described elsewhere [20,24,25]. The procedures for pU1-specific probe labeling and hybridization were the same (see also Section 3.1). The hybridization signals were quantified in a Phosphorimager (Image Analyzer Typhoon FLA 9000; GE Healthcare Bio-sciences, Uppsala, Sweden). The signals of SHwt, SIwt, and their derivatives were normalized by the pU1 transcription level.

The promoter strength of different genes and constructs was evaluated as the ratio between the target plasmid quantity in transfection (a) and the competitor plasmid quantity (b) that suppressed target transcription by 50% (K_0.5_ = a/b). The b values were determined from experimental data presented in plots in Section 3.3.

The secondary RNA structure was predicted using the ViennaRNA Package 2.0 [42] at ViennaRNA Services (http://rna.tbi.univie.ac.at) (accessed on 1 December 2022). The bend.it program [43] was used to predict the bendability and curvature propensity (the window size was 6) at the Bend.it Server (http://pongor.itk.ppke.hu/dna/bend_it.html#/bendit_intro) (accessed on 14 December 2022).

## 3. Results and Discussion

### 3.1. Control Construct Transcribed by RNA Polymerase II

It is imperative to use a suitable control to normalize the quantitative expression data in experiments on cell transfection with genetic constructs. Previously, we used a shrew SOR SINE construct as such a control in cell transfection with 4.5SI or 4.5SH RNA genes [20,23]. Since SOR and these genes are transcribed by pol III, one cannot exclude the mutual influence on their transcription efficiency. Such influence may arise from the competition of SINE and RNA genes for protein factors involved in pol III transcription. To exclude such influences of the control on the transcription of a studied gene, we developed a construct transcribed by pol II. A random 96 nt sequence was generated, and its 5′- and 3′-terminal sequences were slightly edited to allow for a hairpin structure (Figure S1). Our previous experiments suggest that such structures significantly increase the RNA lifetime in the cell [37,44]. The synthetic double-stranded DNA was inserted into pGeneClip, which allows for its transcription by pol II due to the promoter and terminator of the U1 small nuclear RNA gene (Figure S1). Cell co-transfection with this generated plasmid (pU1) and constructs with modified 4.5SI or 4.5SH RNA genes was used in experiments described in the next section. The pU1 transcript was used to normalize the transcriptional level of these genes. The hybridization signal of the pU1 transcript was at least one order of magnitude lower than that of 4.5SI/4.5SH RNA; accordingly, the hybridization signal of pU1 was always measured prior to that of 4.5SI- or 4.5 SH-probes.

### 3.2. Effect of Replacement of Promoter Boxes on Pol III Transcription

**Figure 2:**
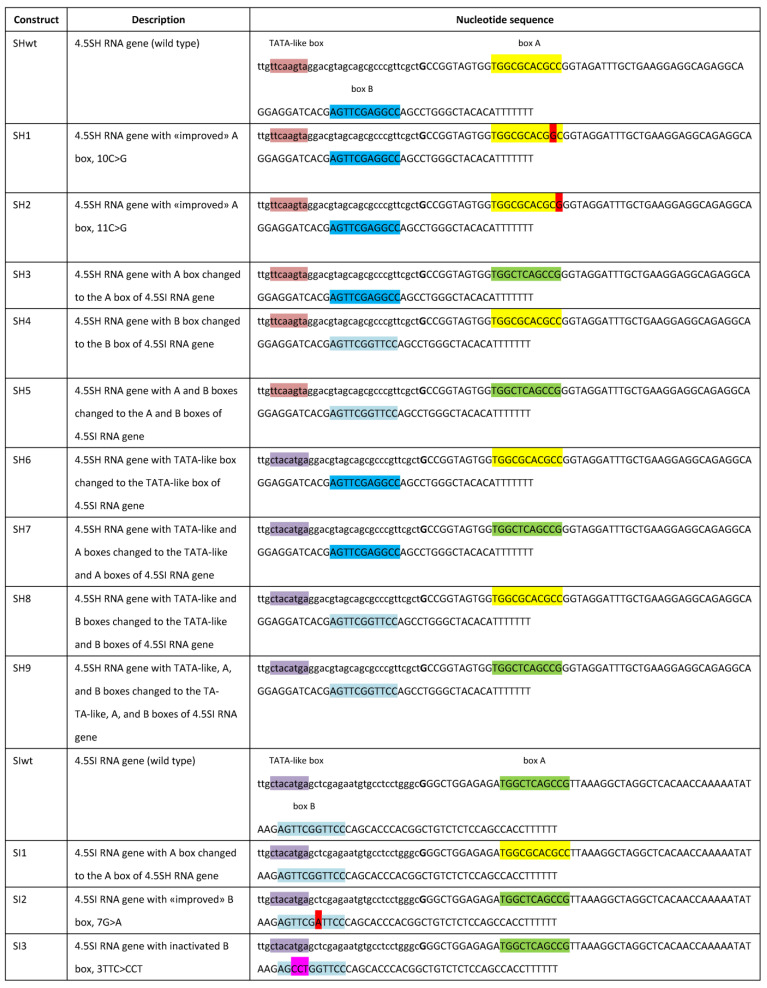
Constructs containing mouse 4.5SH RNA and 4.5SI RNA wild-type genes or their derivatives. Identical pol III promoter boxes A and B, as well as TATA-like boxes, are highlighted in different colors. The 5′-flanking sequences are given in lowercase. The transcribed sequences are given in uppercase. The T_6–7_ block at the end of the genes serves as a pol III transcription terminator. The nucleotides replaced to match the box A or box B consensus sequences are highlighted in red. The trinucleotide in box B marked in magenta was replaced to reduce promoter activity.

**Figure 3:**
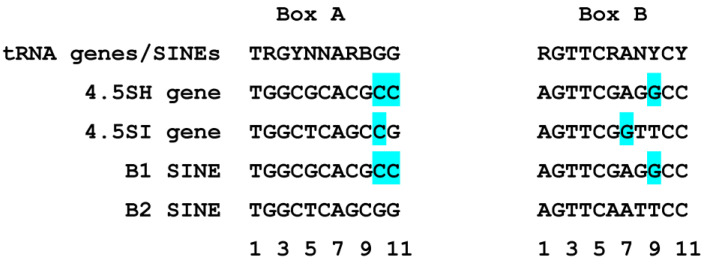
Nucleotide sequences of boxes A and B of 4.5SH and 4.5SI RNA genes, as well as B1 and B2 SINEs. The consensus sequences of boxes A and B of tRNA genes and SINEs [7,9] are shown above, and the nucleotide numbers are shown below. The nucleotides not matching the consensus boxes are highlighted in blue.

Figure 2 presents the nucleotide sequences of the initial murine 4.5SH and 4.5SI RNA genes (SHwt and SIwt, respectively) cloned into pGEM-T and used in transfection experiments. In addition to the sequences with boxes A and B of the internal pol III-directed promoter, these constructs include 34 bp 5′-flanking sequences (lowercase letters in Figure 2) with a TATA-like box at position –31/–24, which is also significant for the transcription of these genes [20,23]. All of these boxes differ distinctly in the 4.5SH and 4.5SI RNA genes. Figure 3 presents the nucleotide sequences of boxes A and B in these two genes in comparison with those of evolutionary-related murine SINEs B1 and B2. The upper line in Figure 3 presents the consensus sequences of boxes A and B deduced from the analysis of 359 human tRNA genes and 175 SINEs of multicellular eukaryotes [7,9]. Notice the nucleotides marked in blue that mismatch with the consensus sequences of human tRNA and SINE. [7]. The effect of three out of five such nucleotides on transcription was experimentally tested. 

**Figure 4:**
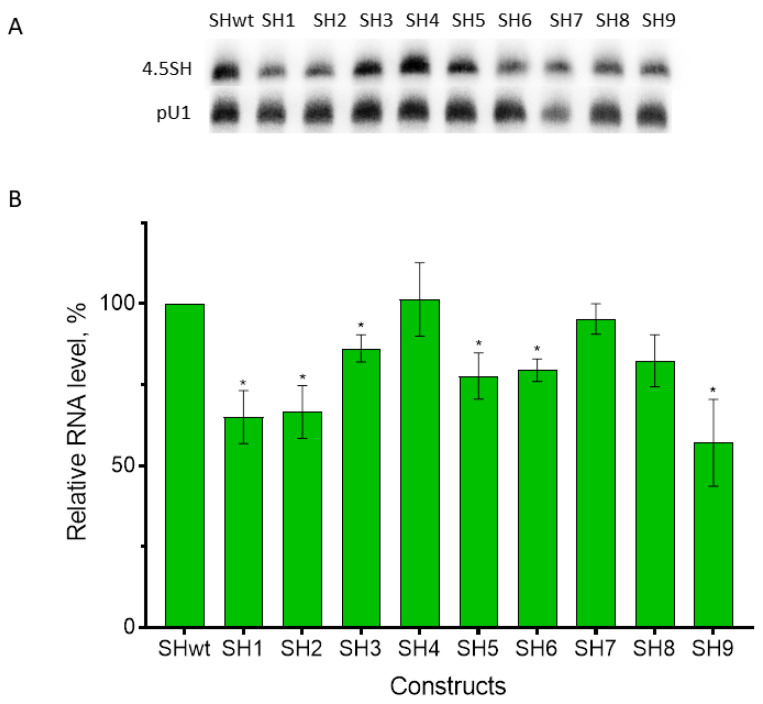
Transcription of 4.5SH RNA gene and its nine derivatives with modified TATA-like, A, or B boxes in the transfected HeLa cells. (**A**) Detection of 4.5SH RNA and its derivatives, as well as pU1 (transfection and loading control) transcripts, via Northern hybridization. (**B**) Quantitative analysis of hybridization data. The level of wild-type 4.5SH RNA (SHwt) is taken as 100%. Error bars, SD, *n* = 3, *t*-test: *—*p* < 0.05.

**Figure 5:**
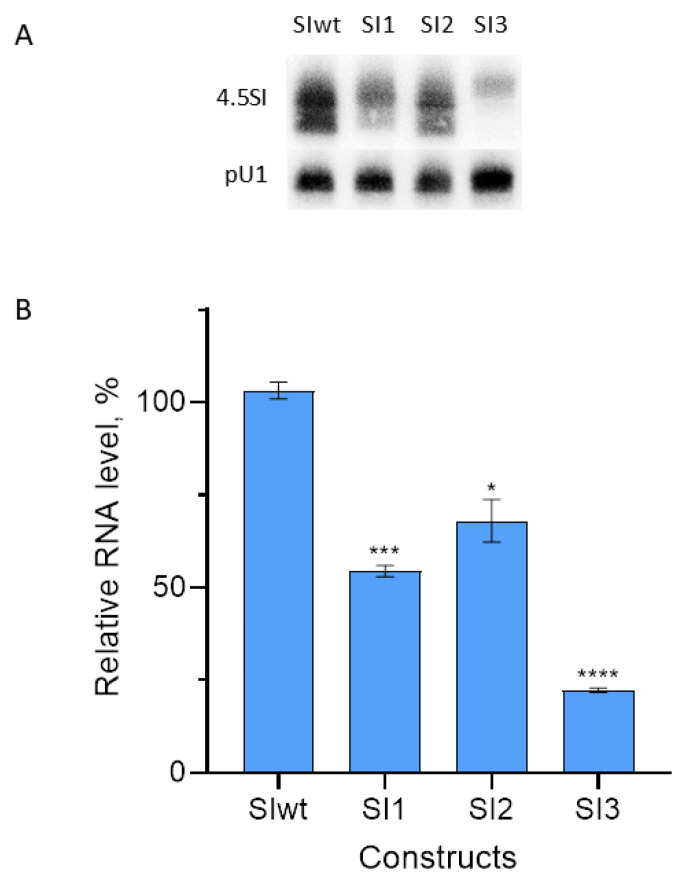
Transcription of 4.5SI RNA gene and its three derivatives with modified A or B boxes in the transfected HeLa cells. (**A**) Detection of 4.5SI RNA and its derivatives, as well as pU1 (control,) via Northern hybridization. (**B**) Quantitative analysis of the hybridization data. The level of wild-type 4.5SI RNA (SIwt) is taken as 100%. Error bars, SD, *n* = 3, *t*-test: *—*p* < 0.05, ***—*p* < 0.005, ****—*p* < 0.0001.

The constructs of the 4.5SH RNA gene with Cs at positions 10 and 11 of box A (Figure 3) were replaced with the canonical Gs (SH1 and SH 2, respectively, in Figure 2). Such substitutions making box A more similar to the consensus could be expected to enhance the transcription efficiency. However, the transfection of HeLa cells with these constructs and Northern blot analysis demonstrated a notable decrease in the transcription level (to 65% relative to the control level, Figure 4). The replacement of G at position 7 of box B in the 4.5SI RNA gene with the canonical A (SI2 in Figure 2) also reduced the transcription efficiency to 67% of control levels (Figure 5). The data obtained indicate a nonrandom appearance of the noncanonical nucleotides in boxes A and B of these genes to optimize their promoters. Arguably, the function of promoters with noncanonical boxes relates to the nucleotide context of the gene or its parts.

A series of 4.5SH RNA gene-based constructs was generated with the A, B, and TATA-like boxes replaced with their counterparts of the 4.5SI RNA gene. All combinations were made with single, double, and triple box substitutions (SH3 to SH9, Figure 2). The transfection of HeLa cells with these constructs and Northern blot analysis (Figure 4) demonstrated no enhanced transcription in either of the cases. The replacement of box B alone (SH4) or the replacement of TATA-like and A boxes (SH7) maintained the 100% transcription level. Presumably, the box B structure has little effect on transcription, whereas TATA-like and A boxes from the same gene (4.5SI) are structurally coordinated to maintain the 100% transcription level. This assumption is supported by a slight transcription reduction (to 80% relative to control levels) after the replacements of box A alone (SH3) or together with box B (SH5), as well as the replacements of the TATA-like box alone (SH6) or together with box B (SH8). These constructs combined heterologous A and TATA-like boxes, and their relative discordance can underlie the reduced transcription efficiency. However, a more significant decrease in the transcription level (to 60% relative to control levels) after the substitution of all three boxes (SH9) is not quite consistent with the presumption. We can only propose the effect of the 4.5SH RNA gene context on heterologous boxes of the 4.5SI RNA promoter.

We also tested the 4.5SI RNA gene construct with box A replaced with that of the 4.5SH RNA gene (SI1 in Figure 2). Such box A substitution substantially (by 50% relative to control) reduced the transcription level (Figure 5). We also generated a construct (SI3) with TTC replaced with CCT in box B ( Figure 2). These three nucleotides are highly conserved among tRNA and SINE genes (Figure 3), which indicates their significance for the box B function. This construct was generated to test the deliberately significant mutation of the pol III promoter. The SI3 transcription level proved to be as low as 20% of that of the wild-type gene SIwt (Figure 5).

It has to be explained why 4.5SH RNA gene-based constructs were prioritized over 4.5SI RNA ones (9 vs. 3, Figure 2). Previously, we demonstrated that 4.5SH is a short-lived RNA with a half-life (T_1/2_) of 20–30 min, while 4.5SI RNA is a relatively long-lived one (T_1/2_ ≈ 20 h). 4.5SI RNA stability in the cell is due to the complementarity of its terminal regions making a long 16 bp double-stranded structure [37]. 4.5SH RNA has no long hairpins, which can cause its rapid degradation in the cell [37]. We observed similarly rapid degradation (T_1/2_ = 20–30 min) of small nuclear RNAs lacking long double-stranded terminal regions or not protected by specific proteins [44]. It is highly unlikely that the replacement of boxes A and/or B in the 4.5SH RNA gene can introduce long double-stranded structures and make its transcript long-lived. Previously, we demonstrated that replacements of extended regions in 4.5SH RNA with those of 4.5SI RNA do not decrease the half-life of such chimeric RNAs in the cell [37]. In all likelihood, the substitutions of boxes A and B in the 4.5SH RNA gene should not reduce the resulting RNA stability. Accordingly, the modifications should not affect the cellular level of 4.5SH RNA by modulating its stability but rather reflect the efficiency of gene transcription. Contrariwise, long substitutions in the 4.5SI RNA gene could likely disrupt the secondary structure of its RNA and shorten its lifetime and level in the cell. Yet, the predicted secondary structures of the modified 4.5SI RNA demonstrated a long double-stranded structure in all of them (Figure S2). This validates our estimates of the transcriptional efficiency of the 4.5SI RNA constructs presented in Figure 5.

### 3.3. Effect of Box Replacement on Promoter Strength

**Figure 6:**
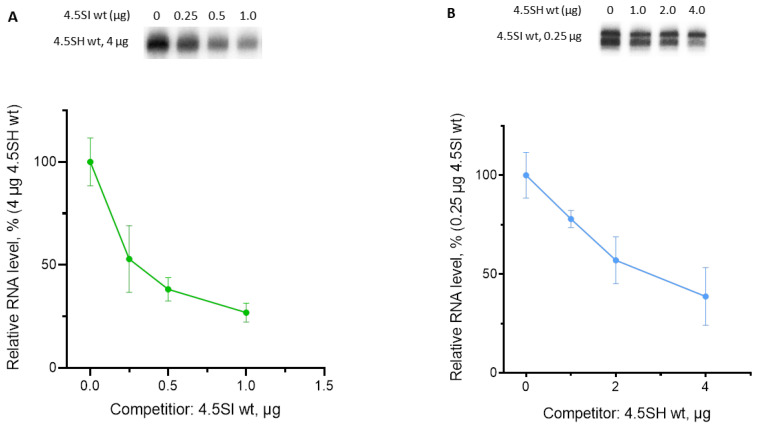
Transcriptional competition of wild-type 4.5SH and 4.5SI genes in transfected HeLa cells. (**A**) Detection of 4.5SH RNA via Northern hybridization. The target plasmid containing the 4.5SH gene (4 μg) was added to each transfection sample, and the amount of the competitor plasmid containing the 4.5SI gene in the transfection mixture is specified above the lanes. (**B**) Detection of 4.5SI RNA via Northern hybridization. The target plasmid containing the 4.5SI gene (0.25 μg) was added to each transfection sample, and the amount of the competitor plasmid containing the 4.5SH gene in the transfection mixture is indicated above the lanes. Plots summarize the Northern hybridization data. The level of 4.5SH RNA or 4.5SI RNA without competition is taken as 100%, and the amount of the competitor is indicated on the X-axis. Error bars, SD, *n* = 3 (see original Northern blot images for three replicates in Figure S4).

We have developed another approach to compare the efficiency of pol III-directed promoters in different genes. It relies on the competition of two co-transfected genes for components of the pol III transcription machinery. These can include RNA polymerase, although the transcription factors TFIIIB and/or TFIIIC are more probable limiting components. The gene of which the promoter has a higher affinity for the transcription factors should be more competitive and suppress the transcription of other genes. This approach demonstrated that the 4.5SI RNA gene promoter is much stronger than that of 4.5SH RNA. A preliminary titration of plasmid amounts added to transfected cells demonstrated that the transcription achieved a plateau at 4 and 0.25 µg for SHwt and SIwt, respectively (Figure S3). These plasmid amounts were used in subsequent competition experiments. Figure 6A illustrates HeLa cell transfections with a constant quantity (4 µg) of the 4.5SH RNA-expressing vector (SHwt, target) but variable quantities of the 4.5SI RNA construct (SIwt, competitor). The cellular level of 4.5SH RNA, evaluated via Northern blot analysis, decreased by 50% after the transfection of as low as 0.3 µg SIwt, which suggests that its promoter is 13 times stronger than that of SHwt (K_0.5_ = 4/0.3 ≈ 13). In the reverse experiment with SIwt as the target and SHwt as the competitor, a 50% decrease in the transcription of 0.25 µg SIwt required about 2.8 µg of SHwt (Figure 6B), which indicates that the SIwt promoter is 11 times stronger than that of SHwt, which is close to the previous estimate. This weakness of the 4.5SH RNA gene promoter relative to that of 4.5SI RNA can be due to specific features of nucleotide sequences of its TATA-like box, as well as boxes A and B (Figure 3). 

The experiments on 4.5SI and 4.5SH RNA gene transcription in transfected cells (Figure 4 and Figure 5) demonstrated no notable difference in the hybridization signals of these RNAs (notice that this comparison is more qualitative than quantitative since different probes specific for 4.5SI and 4.5SH RNAs were used). Apparently, this conflicts with the competition experiments indicating that the 4.5SI RNA gene promoter is 11–13 times stronger than that of 4.5SH RNA. This disagreement can be explained by the large excess of cellular pol III, TFIIIB, and TFIIIC, so that the weaker 4.5SH RNA gene promoter provided for the formation of the transcription initiation complex and active transcription in the absence of competition. The presence of the competitor gene interferes with the initiation complex formation and reduces transcription of the former gene. The promoter efficacy evaluated in competition experiments is referred to as promoter strength below. 

**Figure 7:**
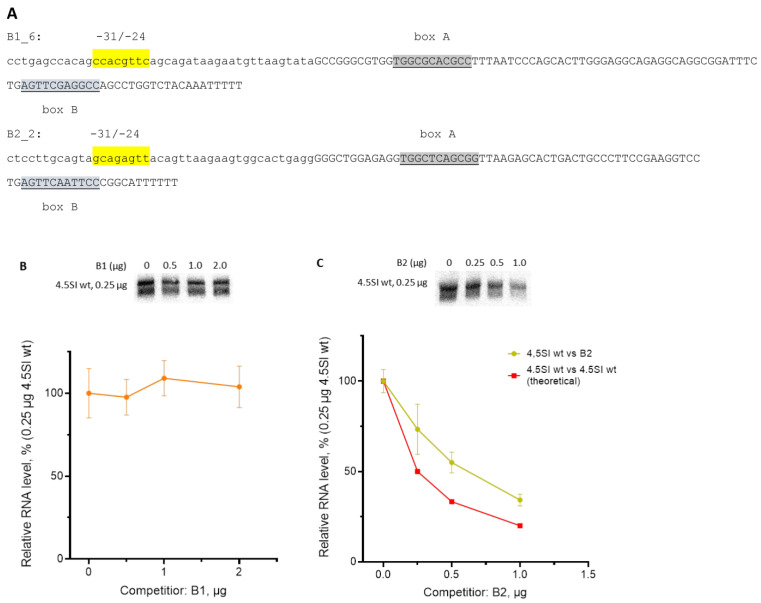
Transcriptional competition of 4.5SI gene with B1 and B2 SINEs in transfected HeLa cells. (**A**) Nucleotide sequences of the constructs containing mouse B1_6 and B2_2 copies. SINEs were truncated downstream of boxes B, and a transcription terminator T_5–6_ was added to the end. The SINE and 5′-flanking nucleotide sequences are shown in uppercase and lowercase, respectively. The nucleotides at positions −31/−24 are highlighted in yellow. Boxes A and B are underlined and highlighted in grey. (**B**) Transcription of 4.5SIwt with different amounts of the B1_6 construct (competitor). (**C**) Transcription of 4.5SIwt with different amounts of the B2_2 construct (competitor). The plots below summarize Northern hybridization data. The red line in (**C**) shows competition of 4.5SIwt with a hypothetical gene with a promotor of the same strength as that of the 4.5SI RNA gene promotor. Error bars, SD, *n* = 3 (see original Northern blot images for three replicates in Figure S4).

To evaluate the potential of the approach, we conducted experiments with the 4.5SI RNA gene as the target and B1 or B2 SINEs as competitors. As mentioned previously, the 4.5SH and 4.5 SI RNA genes originated from B1 and B2 SINEs, respectively. As a result, the A and B boxes are identical in 4.5SH RNA and B1 and highly similar in 4.5SI RNA and B2 genes (Figure 3). Previously, we tested the transcription of randomly selected murine B1 and B2 copies (10 and 9 copies, respectively); however, two of them were studied in detail, B1_6 and B2_2 [20]. These copies demonstrated modest transcriptional activity: as low as 15 and 40% of the corresponding copies with the top transcription, respectively. The distinctions between the copies underlying their different transcriptional efficiencies reside in the 5′-flanking sequences within the −31 to −24 region [20]. Here, we used these B1_6 and B2_2 copies as competitors (Figure 7A) in co-transfection experiments with 0.25 µg of SIwt. Even an 8-fold excess of B1_6 failed to suppress SIwt transcription (Figure 7B), thus indicating the weakness of the B1_6 promoter. This can be attributed to the features of the box A nucleotide sequence (Figure 3) and the abundance of C and G in the –31 to –24 region (5 out of 8; Figure 7A), which makes it a poor TATA-like box. Conversely, the B2_2 construct successfully competed with SIwt (Figure 7C); 0.6 µg B2_2 decreased the transcription of 0.25 µg SIwt by 50% (K_0.5_ = 0.25/0.6 ≈ 0.4). Still, the B2_2 promoter was weaker (2.4 times) than that of the 4.5SI RNA gene, which can be attributed to the nucleotide sequence imperfections in the –31 to –24 region (boxes A and B in B2_2 perfectly match the consensus sequences).

**Figure 8:**
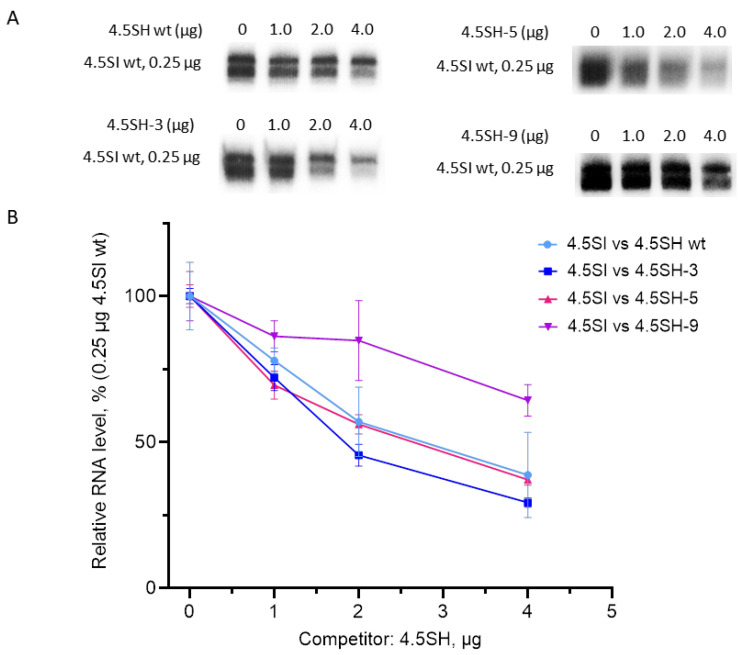
Transcriptional competition of 4.5SI gene with 4.5SH gene derivatives in transfected HeLa cells. (**A**) Northern blot detection of 4.5SI RNA. The target plasmid containing the 4.5SI gene (0.25 μg) was added to each transfection sample, and the amount of the competitor plasmid containing the 4.5SH gene (SHwt) or its derivatives (SH-3, SH-5, and SH-9) in the transfection mixture are indicated above the lanes. (**B**) Quantitative analysis of the Northern blot data. The level of 4.5SI RNA without competition is taken as 100%, and the amount of the competitors is indicated on the X-axis. Error bars, SD, *n* = 3 (see original Northern blot images for three replicates in Figure S4).

Finally, we conducted competition experiments with 4.5SH RNA gene constructs with box A (SH3), boxes A and B (SH5), and all three pol III promoter boxes (SH9) replaced with their counterparts of the 4.5SI RNA gene as competitors. About 1.8 µg of SH3 suppressed the target (SIwt) transcription by 50% (K_0.5_ = 0.25/1.8 ≈ 0.14) (Figure 8). Thus, the box A substitution increased the promoter activity roughly 1.5-fold. Simultaneous substitution of boxes A and B (SH5) restored the promoter strength to the level of the original 4.5SH RNA gene (Figure 8). The TATA-like box substitution in addition to boxes A and B substantially reduced the promoter strength; the co-transfection of 4 µg SH9 decreased SIwt transcription to as low as 65% of the control level (Figure 8). Notice that these data agree with the clearly decreased SH9 transcription level in the above transfection experiment without competition (Figure 4).

These results were surprising because we expected the boxes of a strong promoter from the 4.5SI RNA gene to create a strong promoter when transferred to the 4.5SH RNA gene. This was not the case, and slight promoter enhancement was observed only after the replacement of box A alone. The pronounced promoter reduction after the simultaneous substitution of all three boxes was especially unexpected. Apparently, the boxes of the 4.5SI RNA gene within another gene (4.5SH) with “foreign” nucleotide sequences proved to be less efficient ligands of TFIIIB and TFIIIC and thus weaker promoters. Accordingly, the nucleotide context can modulate the function of pol III-directed promoters, and the nucleotide sequences of all three boxes were optimized for the surrounding gene sequences in evolution.

The mechanism by which sequences outside of promoters can modulate their activity remains unclear. DNA bendability can be one of the factors of promoter function. There are indications that the bendability of the 5′-flanking sequences of genes can influence pol III transcription [12]. When the TATA-binding protein (TBP) in the TFIIIB complex interacts with TATA or TATA-like box, it sharply bends DNA at the binding site [8,10]. Statistical analysis of 5′-flanking sequences of yeast tRNA genes revealed an elevated DNA bendability of the TATA-like box area and a reduced bendability of the downstream area [12]. The bend.it program was used to analyze the SHwt, SIwt, and SH9 constructs and demonstrated that (i) expectedly the TATA-like box and the downstream region in SIwt have high and low bendability, respectively; and (ii) the TATA-like box in SHwt has no elevated bendability (Figure S5). Moreover, the 4.5SI RNA gene has a region (A_5_) between boxes A and B with very low bendability (around position 80 in Figure S5, SIwt). It is not improbable that these features of the 4.5SI and 4.5SH RNA genes modulate their promoter function.

Another possible factor of pol III-directed promoter activity is the distance between boxes A and B. They are spaced by 32 and 40 bp in the 4.5SI and 4.5SH RNA genes, respectively (Figure 2). This distance can significantly vary between pol III-transcribed genes. The analysis of 359 human tRNA genes allowed us to divide them into two groups based on the distance between boxes A and B: 32–34 and 41–43 bp; the former group includes the majority (86%) of genes (Figure S6). There are genes with a longer spacer, e.g., 52 bp in the 7SL RNA gene [32,45]. One can propose that the nucleotide sequences of all three boxes of the pol III promoter are tuned to a particular gene sequence, as well as the distance between boxes A and B. Thus, the boxes transferred to a different gene with originally different distances between them can make up a weaker promoter.

To our knowledge, the observed effect of the gene nucleotide context of pol III promoter efficiency has not been described previously, possibly due to the following. First, the pol III promoter boxes were not shuffled between genes. Second, we experimented with genes of different origins (7SL RNA-derived 4.5SH RNA and tRNA-derived 4.5SI RNA). Third, we used a new approach based on transcriptional competition between genes to evaluate their promoter strength.

## 4. Conclusions

The 4.5SH RNA gene has Cresidues at positions 10 and 11 of box A, which is uncommon for the pol III-directed promoter. Their replacement with the Gs found in the box A consensus sequence decreased rather than increased the transcription efficiency in transfected cells. Box B of the 4.5SI RNA gene has a non-canonical G at position 7, and its replacement with the canonical A also clearly reduced the gene transcription efficiency. These data indicate that specific nucleotides in boxes A and B can increase the efficiency of the pol III-directed promoter in particular genes. Replacements of A, B, or TATA-like boxes alone or boxes A and B together in the 4.5SH RNA gene with those of the 4.5SI RNA gene either had no effect or decreased the transcription efficiency. This indicates promoter activity dependence on the gene nucleotide context. A method was developed to compare promoter activities based on transcriptional competition between co-transfected genes. The 4.5SH RNA gene promoter proved to be 12 times weaker than that of the 4.5SI RNA gene. The murine B1 SINE promoter also proved to be weak, while that of B2 SINE was strong. Simultaneous replacement of the three boxes of a weak promoter of the 4.5SH RNA gene with those of the strong 4.5SI RNA gene promoter decreased, rather than increased, its activity. One can speculate that promoters of a gene transferred to a different gene with heterologous nucleotide sequences largely lost their capacity to bind the transcription machinery and thus their promoter strength. Consequently, the nucleotide context of genes can modulate the function of internal pol III-directed promoters. In all likelihood, the promoter efficiency also depends on the distance between boxes A and B, as well as DNA bendability in the TATA-like box area. In addition, certain nucleotides outside of the three boxes can also contribute to promoter strength. So far, the transcription of genes with type 2 promoters by pol III was considered to be governed solely by A, B, and TATA-like boxes. The data obtained in this work indicate that the function of these boxes is not self-sufficient and depends on the nucleotide environment.

## Data Availability

Not applicable.

## Supplementary Materials

The following supporting information can be downloaded at: https://www.mdpi.com/article/10.3390/genes14040802/s1, Figure S1: Structure of pU1; Figure S2: Predicted secondary structures of transcripts of the 4.5SI gene (SIwt) and its derivatives (constructs SH1, SH2, and SH3); Figure S3: Titration of 4.5SHwt and 4.5SIwt plasmids; Figure S4: The original images of Northern blot hybridization of three transfection replicates; Figure S5: Curvature propensity and bendability plot of DNA sequences of SIwt, SHwt, and SH9; Figure S6: Lengths of spacers between boxes A and B of RNA polymerase III internal promotors in 359 human tRNA genes.
