# Supplementary material for: Nucleotide Context Can Modulate Promoter Strength in Genes Transcribed by RNA Polymerase III
Source: Genes (Basel). 2023 Mar 27;14(4):802. doi: 10.3390/genes14040802 (PMC10137851; doi:10.3390/genes14040802)
Supplement: Supplementary file 1 [file genes-14-00802-s001.zip › Figure S1.pdf]

1 20 40 60
TCTC**G**GCATTGACTAGTTCGAGGTAATCACGATGATAGCTCTGTACGTATGATAATACTGATCGTAGGTC
80
TGCTCAGTAGCAAGCGAACTAGTCAATGCT 3’

Figure S1. Structure of pU1. A random 96 bp nucleotide sequence was cloned into pGeneClip for its transcription by RNA-polymerase II governed by the promotor and terminator of the U1 RNA gene in the vector. The transcription starts with G at position 1. The complementary regions of the insertion that form a double-stranded structure in the transcript are underlined. The complementary sequence used for the PCR primer to synthesize the labeled probe is highlighted in yellow.
