# Supplementary material for: Nucleotide Context Can Modulate Promoter Strength in Genes Transcribed by RNA Polymerase III
Source: Genes (Basel). 2023 Mar 27;14(4):802. doi: 10.3390/genes14040802 (PMC10137851; doi:10.3390/genes14040802)
Supplement: Supplementary file 1 [file genes-14-00802-s001.zip › Figure S2.pdf]

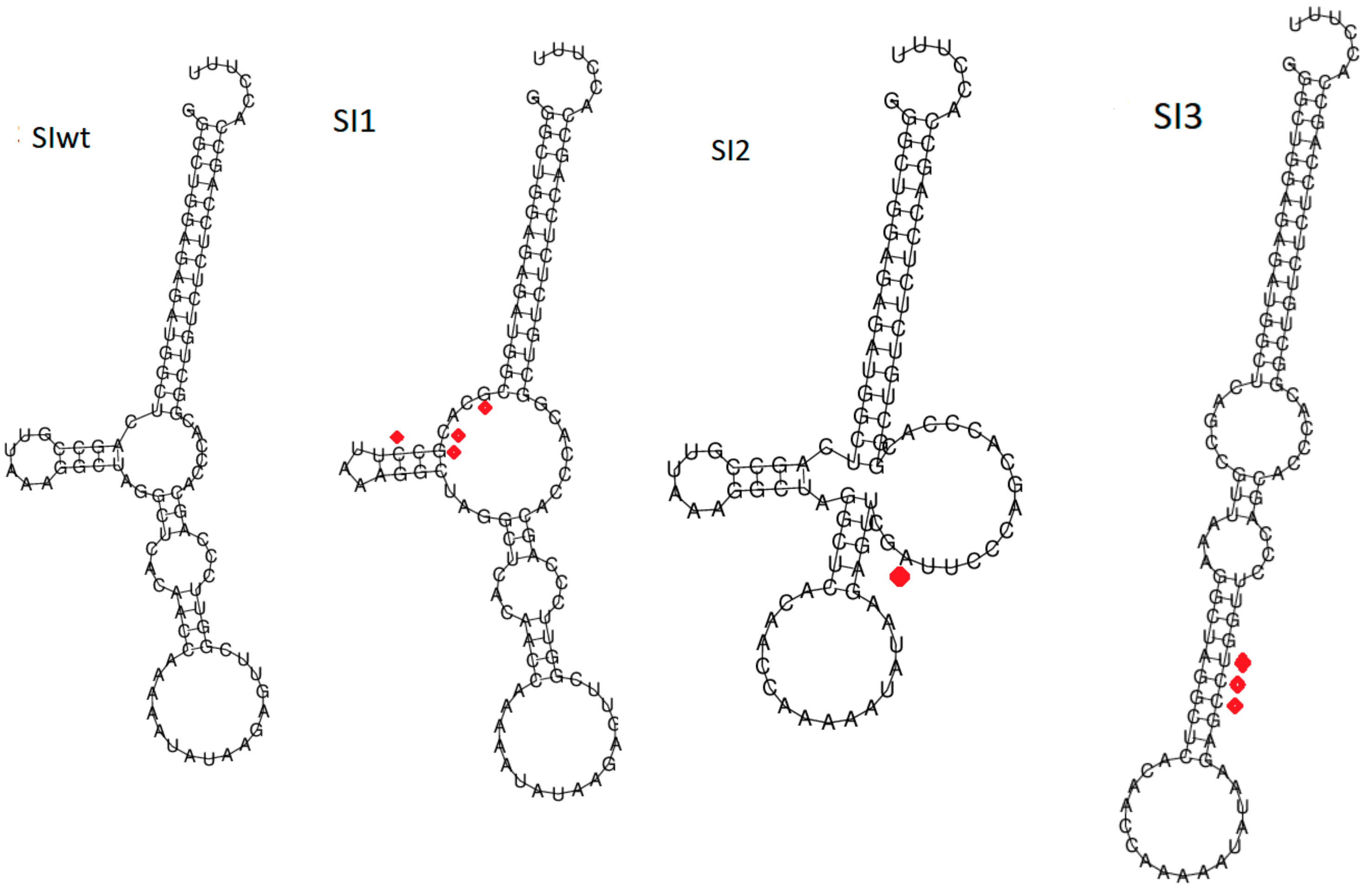

Figure S2. Predicted secondary structures of transcripts of 4.5SI gene (SIwt) and its derivatives (constructs SH1, SH2, and SH3). Modified nucleotides are marked with red diamonds. Note a long stem formed by terminal regions in all transcripts.
