# Supplementary material for: Nucleotide Context Can Modulate Promoter Strength in Genes Transcribed by RNA Polymerase III
Source: Genes (Basel). 2023 Mar 27;14(4):802. doi: 10.3390/genes14040802 (PMC10137851; doi:10.3390/genes14040802)
Supplement: Supplementary file 1 [file genes-14-00802-s001.zip › Figure S3.pdf]

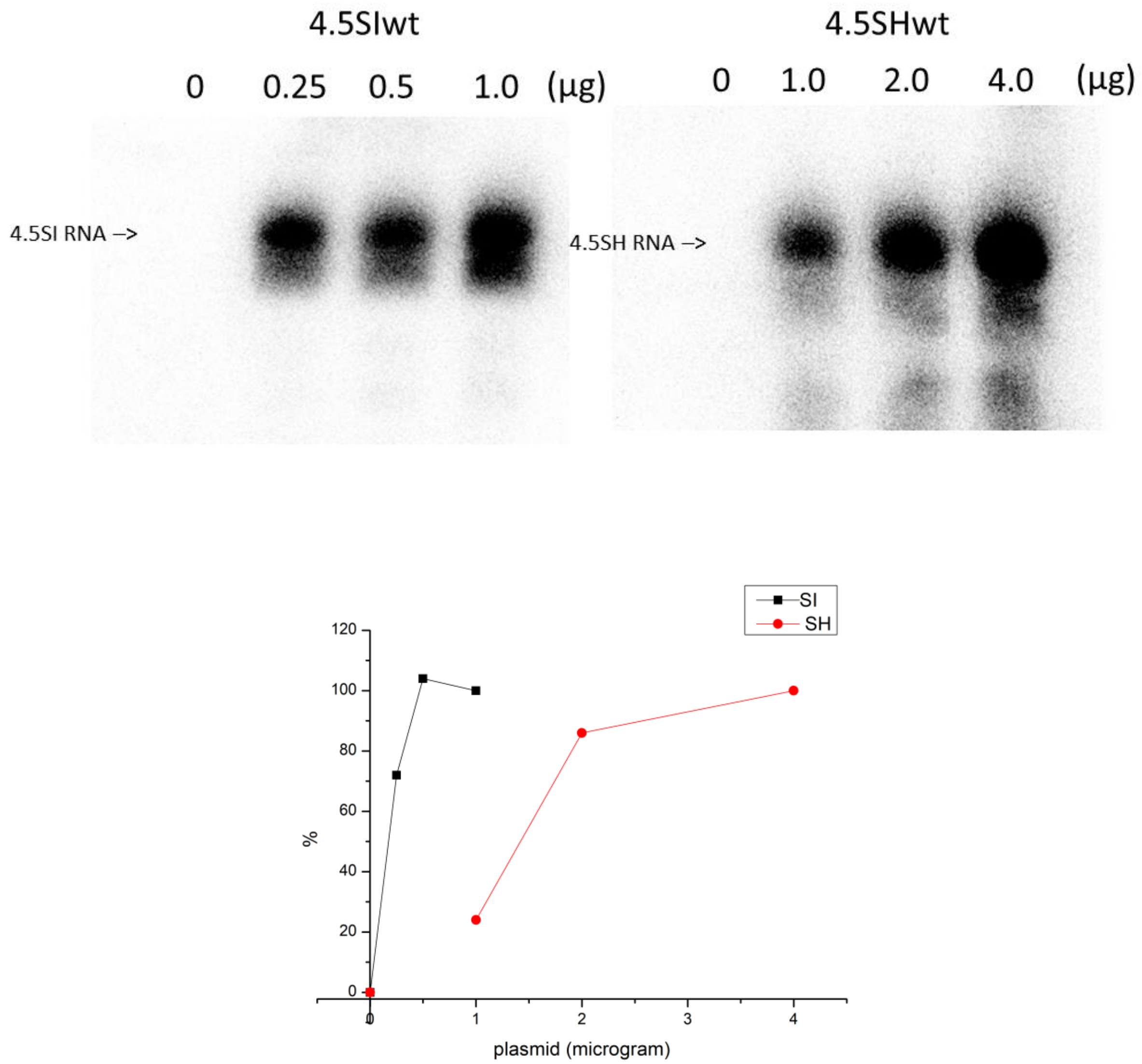

Figure S3. Titration of 4.5SHwt and 4.5SIwt plasmids. Northern blot hybridization detection of RNA transcribed from the indicated amounts of the plasmids transfected to HeLa cells. The graphs demonstrate the change in the hybridization signal depending on the amount of plasmid added. (N = 3).
