# Supplementary material for: Nucleotide Context Can Modulate Promoter Strength in Genes Transcribed by RNA Polymerase III
Source: Genes (Basel). 2023 Mar 27;14(4):802. doi: 10.3390/genes14040802 (PMC10137851; doi:10.3390/genes14040802)
Supplement: Supplementary file 1 [file genes-14-00802-s001.zip › Figure S4.pdf]

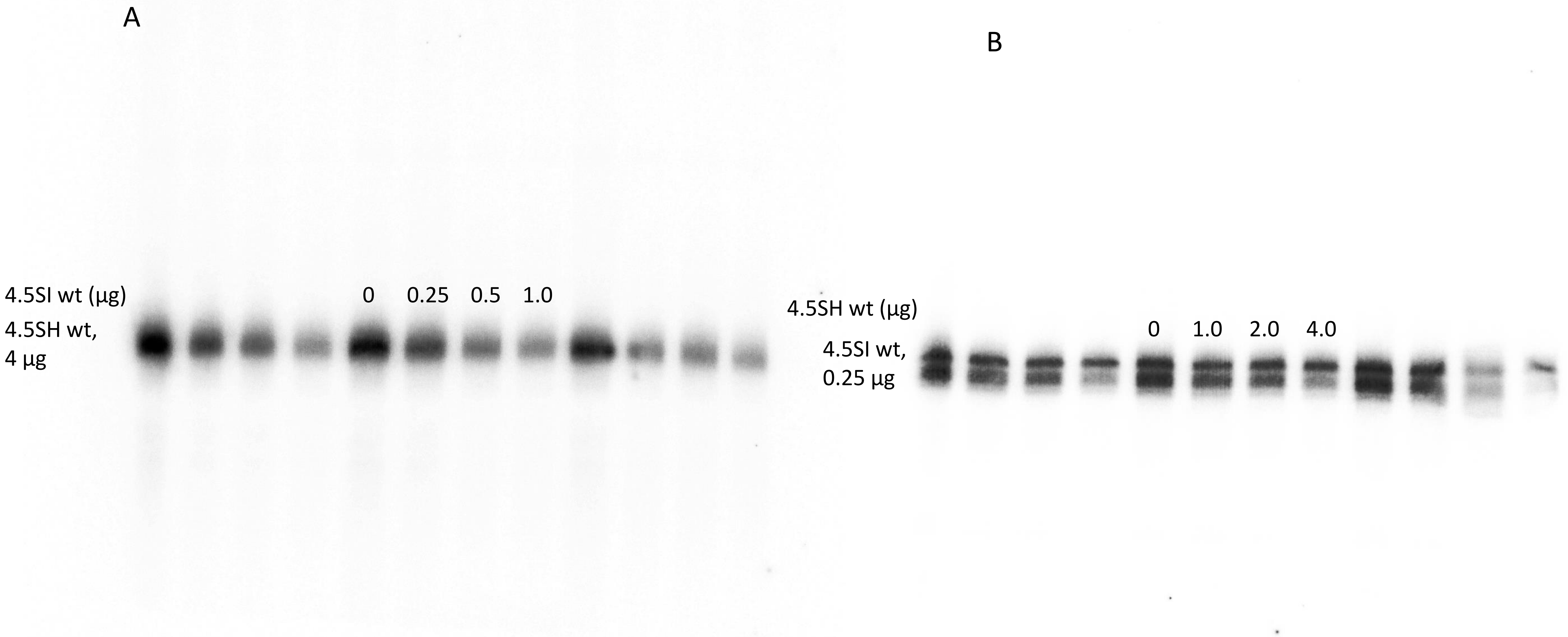

Figure S4. The original images of northern blot hybridization of three transfection replicates. (For figures 6A and 6B in the main text).

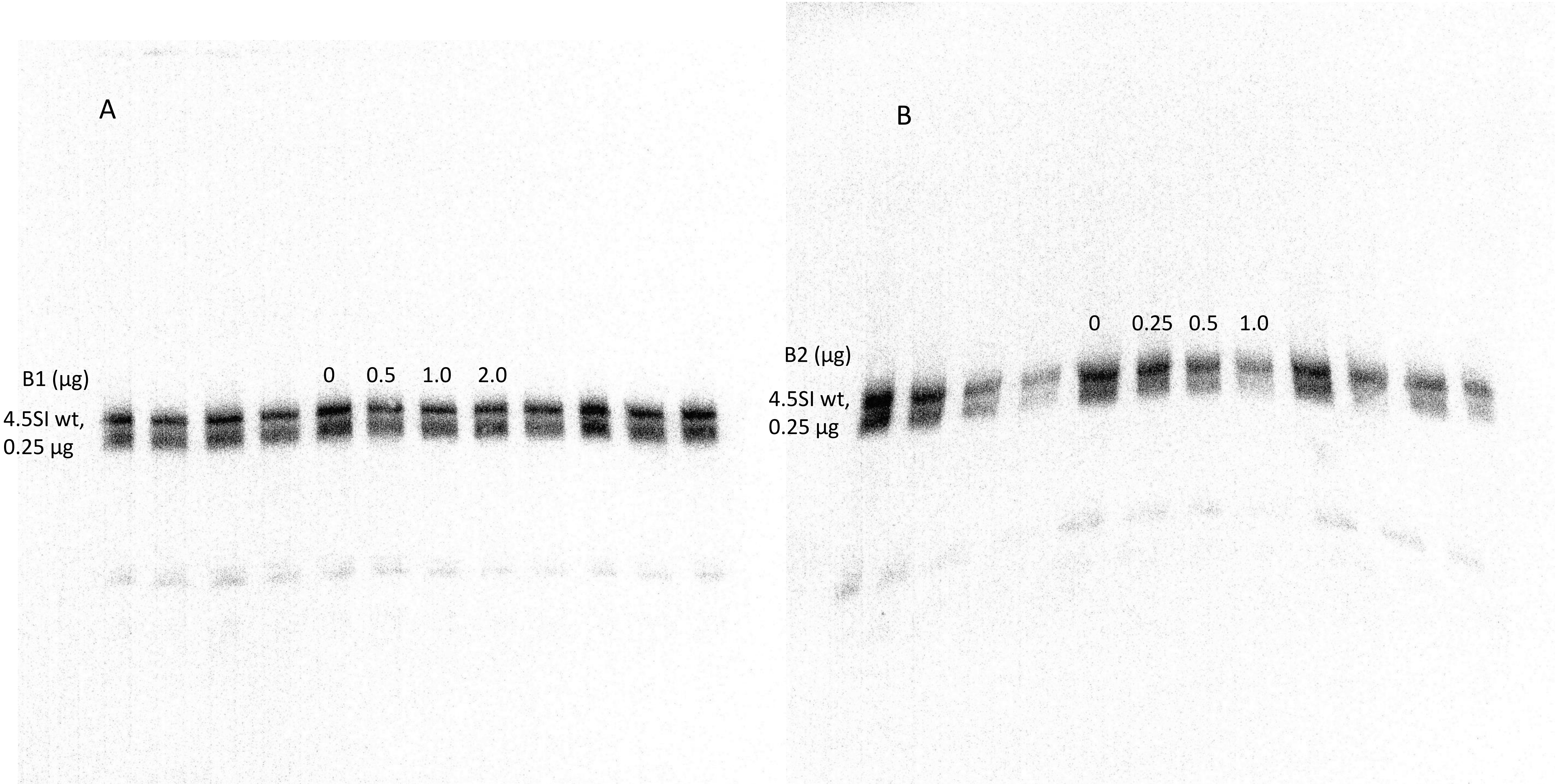

Figure S4, continued. The original images of northern blot hybridizations of three transfection replicates. (For figures 7A and 7B in the main text).

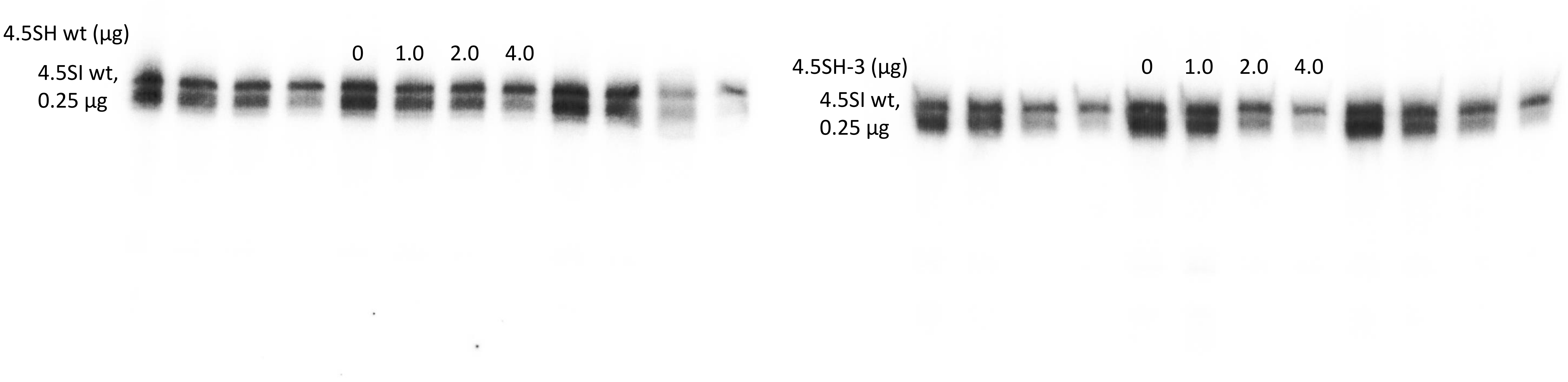

Figure S4, continued. The original images of northern blot hybridizations of three transfection replicates. (For two left blots in figure 8 of the main text).

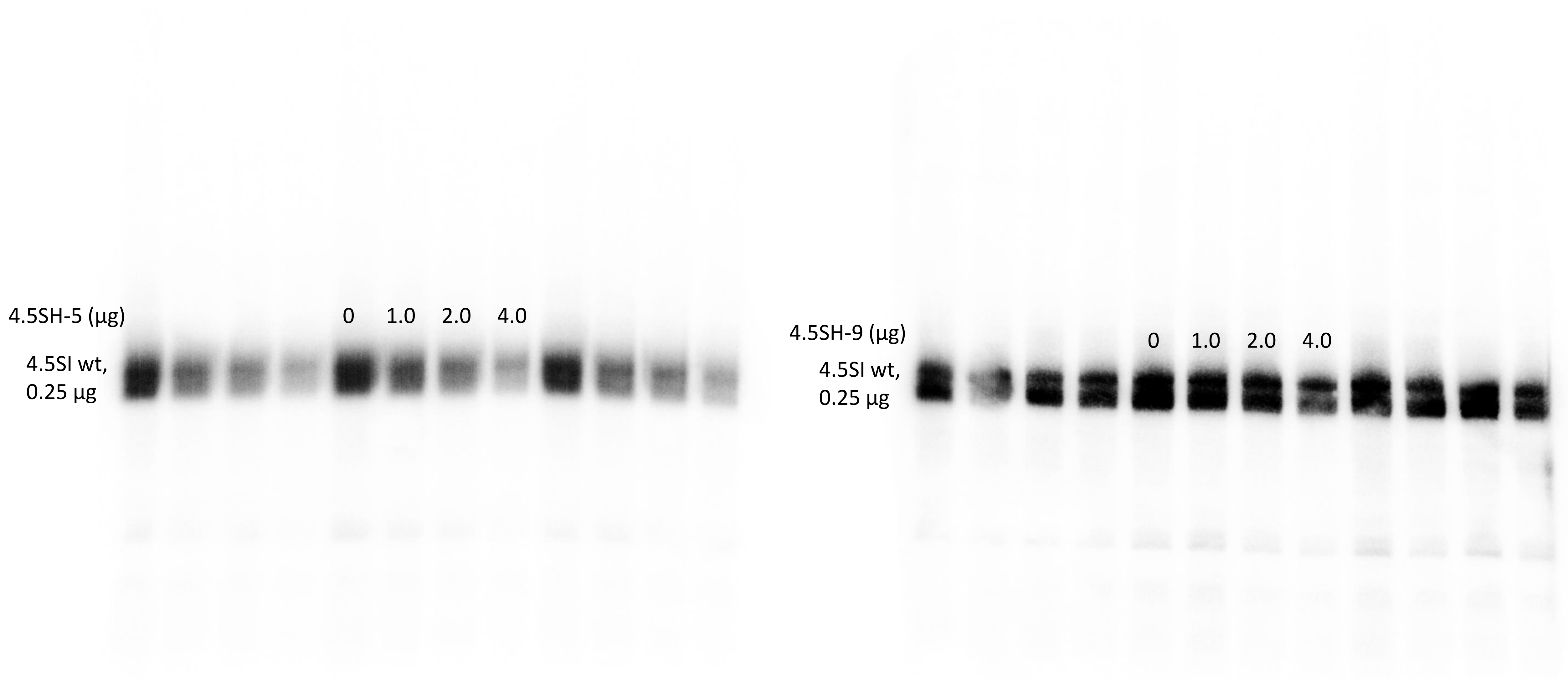

Figure S4, continued. The original images of northern blot hybridizations of three transfection replicates. (For two right blots in figure 8 of the main text).
