# Supplementary material for: Nucleotide Context Can Modulate Promoter Strength in Genes Transcribed by RNA Polymerase III
Source: Genes (Basel). 2023 Mar 27;14(4):802. doi: 10.3390/genes14040802 (PMC10137851; doi:10.3390/genes14040802)
Supplement: Supplementary file 1 [file genes-14-00802-s001.zip › Figure S5.pdf]

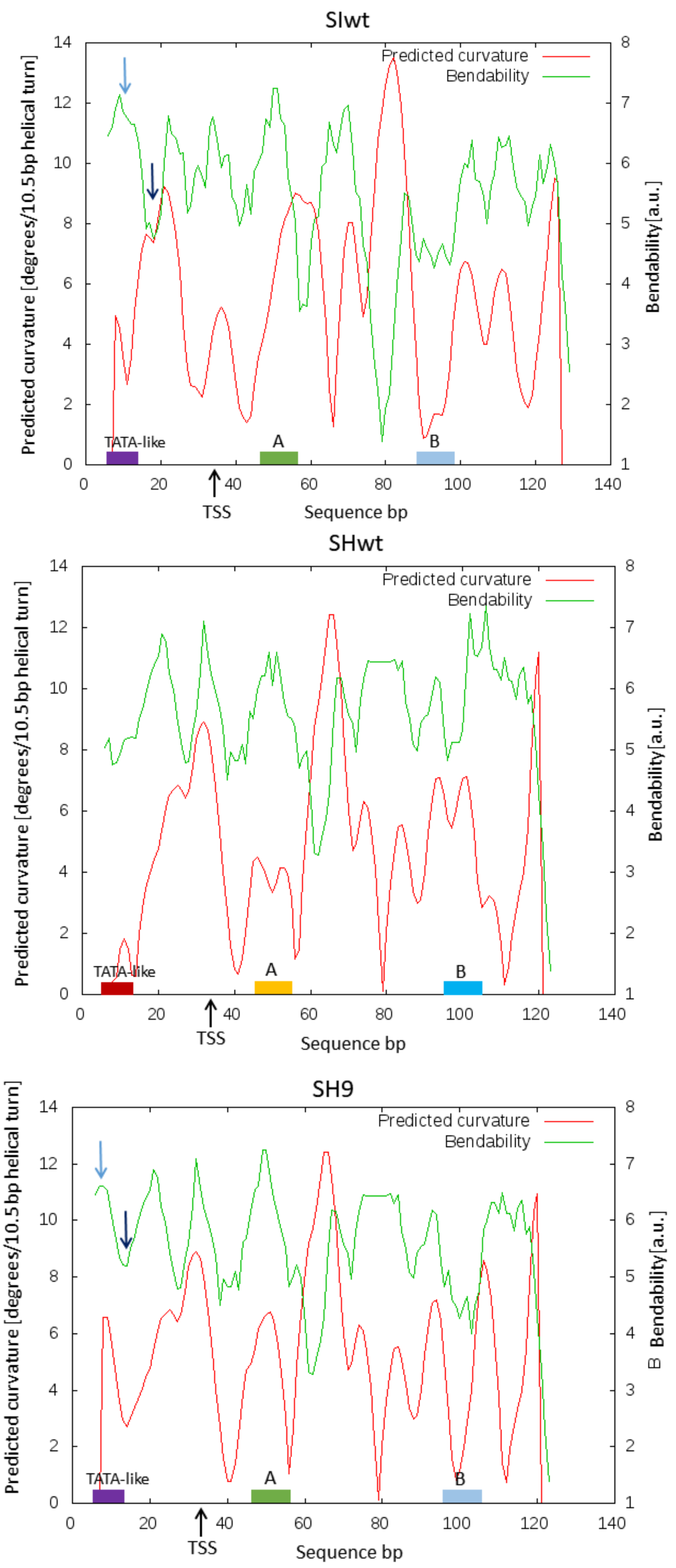

Figure S5. Curvature propensity and bendability plot of DNA sequences of SIwt, SHwt, and SH9 (see nucleotide sequences in Figure 2). Predicted curvature and bendability are shown in red and green, respectively. TATA-like, A, and B boxes are shown as rectangles on the X-axis. Positions of transcription start sites (TSSs) are indicated with black arrows. The peak of bendability corresponding to the TATA-like box in SIwt and SH9 constructs is marked by blue arrows. The next sites in order of bendability are marked by dark blue arrows.
