# Supplementary material for: Nucleotide Context Can Modulate Promoter Strength in Genes Transcribed by RNA Polymerase III
Source: Genes (Basel). 2023 Mar 27;14(4):802. doi: 10.3390/genes14040802 (PMC10137851; doi:10.3390/genes14040802)
Supplement: Supplementary file 1 [file genes-14-00802-s001.zip › Figure S6.pdf]

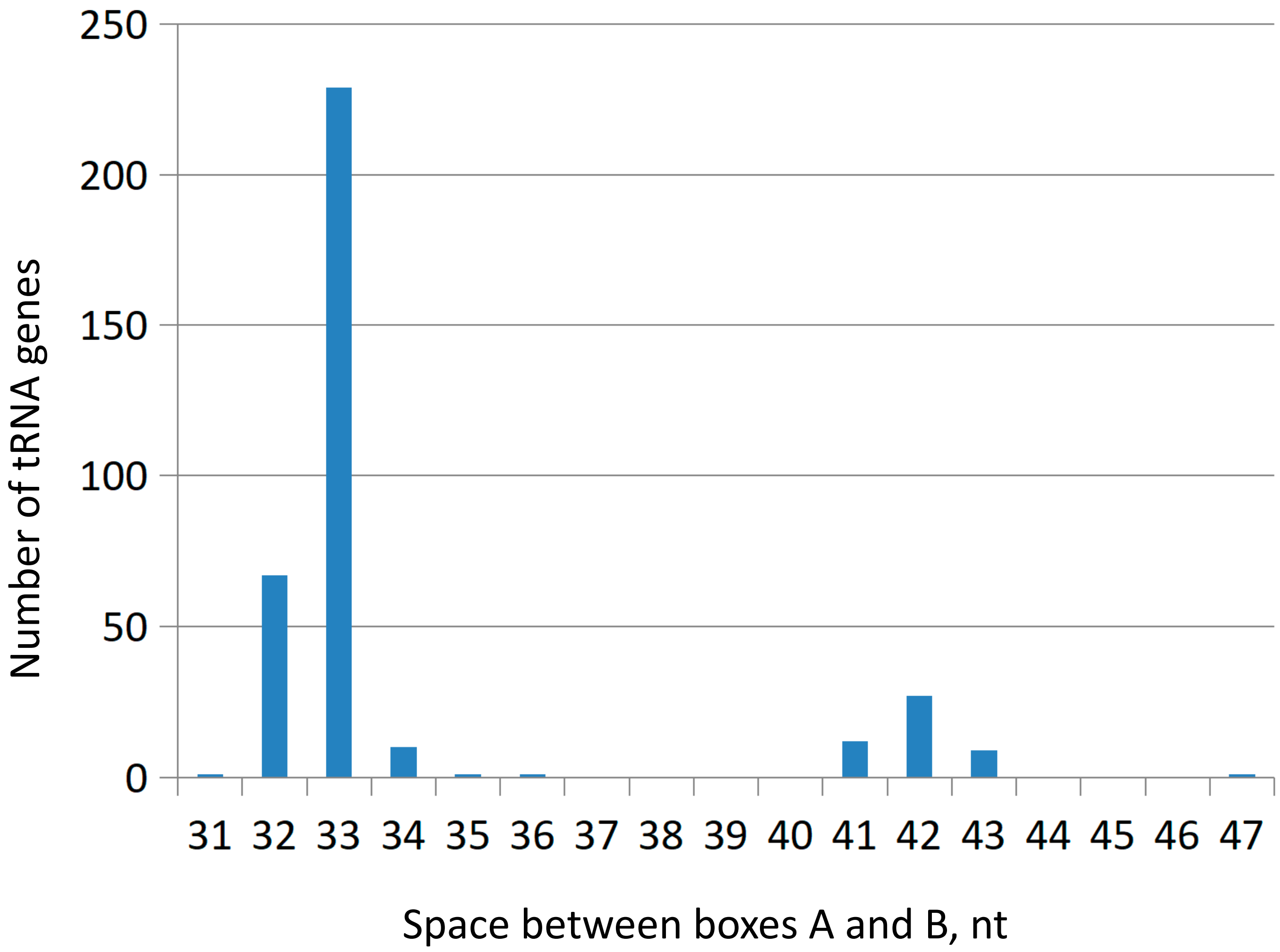

Figure S6. Lengths of spacers between boxes A and B of RNA polymerase III internal promotors in 359 human tRNA genes.
